# Impact of drug substitution on cost of care: an example of economic analysis of cetuximab versus panitumumab

**DOI:** 10.1186/s12962-018-0132-9

**Published:** 2018-11-12

**Authors:** Yifan Xu, Joel W. Hay, Afsaneh Barzi

**Affiliations:** 1 Schaeffer Center for Health Policy and Economics, University of Southern California, 635 Downey Way, Suite 310, Los Angeles, CA 90089 USA; 2 Norris Comprehensive Cancer Center, University of Southern California, 1441 Eastlake Ave. Suite 3440, Los Angeles, CA 90033 USA

**Keywords:** Cost-effectiveness, Economic evaluation, Quality-adjusted life years, Panitumumab, Cetuximab, mCRC

## Abstract

**Background:**

The alarming increase in the cost of cancer care is forcing all stakeholders to re-evaluate their approach to treatment. Drugs are the main contributor to the cost. To evaluate the significance of drug substitution on the cost of care we assessed the economic value of panitumumab vs. cetuximab in chemo-refractory metastatic CRC (mCRC) with wild-type KRAS from a US societal perspective.

**Methods:**

We developed a Markov model with three health states: progression-free, progressive, and death. We calculated the transition probabilities between states using the ASPECCT trial report and US life tables. Costs of drug and administration were based on the Medicare reimbursement rates. Published data were used for cost of toxicities and utilities. All costs were converted to 2017 US dollars. The model used quality-adjusted life-years (QALYs) to measure health outcomes for each treatment option.

**Results:**

Panitumumab and cetuximab produced 0.45 QALYs at a per patient cost of $66,006 and $71,956, respectively. The incremental net monetary benefit of panitumumab compared to cetuximab is $5237 under a societal willingness-to-pay threshold of $150,000. The model showed robustness to one-way sensitivity analyses and various alternative scenarios and was found to be most sensitive to the cost of cetuximab.

**Conclusions:**

Panitumumab can lower the cost of care without impacting outcomes in chemo-refractory mCRC settings. This finding provides a strong argument to consider panitumumab in lieu of cetuximab in these patients.

## Background

The number of agents for treatment of cancer is increasing and so is the cost of cancer care. Up until recently, the number of equivalent agents for every indication were very limited and thus substitution of agents was not an option. However, development of immune check point inhibitors with their broad efficacy is changing this landscape. Currently several immune check point inhibitors with similar efficacy and indication have regulatory approval around the world and the numbers are increasing rapidly. We evaluated the drug substitution for panitumumab and cetuximab in patients with metastatic colorectal cancer to illustrate that incremental net monetary benefit can be the differentiating factor in drugs with comparable efficacy and toxicity.

In United States, colorectal cancer is one of the leading causes of cancer-related death and a common cancer among men and women. In 2016, an estimated 134,490 new cases and 49,190 deaths were attributed to colorectal cancer [1]. Among newly diagnosed colorectal cancers, up to 20% of patients present with metastatic colorectal cancer (mCRC) and roughly 40% of patients with non-metastatic disease will develop metastatic disease in their lifetime. The 5-year survival rate for the mCRC population remains low at 12.5% [2, 3].

There are a number of different drugs currently approved and used in the United States and around the world with significant antitumor activity in mCRC. These include anti-Epidermal Growth Factor Receptor (EGFR) antibodies, cetuximab and panitumumab, which have been shown to improve outcomes among mCRC patients in different lines of therapy [4]. KRAS (Kirsten rat sarcoma) mutation is identified as a predictive marker of poor response to EGFR antibodies, and therefore, cetuximab and panitumumab are administered only in KRAS wild type carriers. Other molecular markers, such as NRAS, BRAF, and EGFR mutations as well as the location of the tumor, also predict treatment outcomes and allow for limited delivery of treatments to the most susceptible patient populations. Although efforts to select suitable treatments for patients based on their biomarker profile improves the treatment value, these treatments are very costly even in highly selected populations [5, 6].

The ASPECCT trial (NCT01001377) was a head-to-head, non-inferiority phase 3 randomized trial that compared panitumumab and cetuximab in mCRC patients with wild-type KRAS who had failed (i.e. disease progression) or were intolerant to irinotecan-based and oxaliplatin-based therapy. The trial enrolled a total of 1010 subjects [7]. Based on the ASPECCT final report, these two agents have similar clinical benefit and safety profiles. While these agents have comparable efficacy and toxicity profiles, they have different administration schedules and costs. In our study, we considered the clinical efficacy, direct costs (including drug costs, costs of administration and adverse events management, costs of end-of-life care), indirect costs (care-giver costs), and utilities to project the cost-effectiveness of one agent with the other.

We used data reported in ASPECCT to evaluate the cost-effectiveness of panitumumab and cetuximab as monotherapy in chemo-refractory mCRC patients with wild-type KRAS from a US societal perspective. All monetary results are reported in 2017 US dollars.

## Methods

A Markov model consisting of three health states (i.e., progression-free state, progressive state, and death) was constructed in Microsoft Excel (Table 1 for model parameters). Transition probabilities between states were calculated using survival data from the ASPECCT trial, a head-to-head, non-inferiority phase 3 randomized trial that compared panitumumab and cetuximab in mCRC patients with wild-type KRAS who had failed (i.e. disease progression) or were intolerant to irinotecan-based and oxaliplatin-based therapy. The model assumed a 2-year time horizon, given the limited life expectancy of the target population. Patients were assumed to take either panitumumab or cetuximab until disease progression. Upon disease progression, all patients were assumed to receive either regorafenib or TAS-102 with equal probability, before receiving best supportive care.

**Table 1:** Model parameters

	Panitumumab	Cetuximab	Range tested for sensitivity analysis	References
Survival outcomes (months)				
Median PFS	4.1	4.4	95% CI	[7]
Median OS	10.4	10	95% CI	[7]
Cost				
Drug acquisition	$4224^a^	$1113^b^	± 20%	[10]
Chemotherapy administration	$272	$330	± 20%	[12]
AEs management (per event)			± 20%	
Skin rash	$5484			[13]
Hypomagnesemia	$7654			[13]
Hypokalemia	$1605			[15]
Infusion reaction	$2138 (Grade 1 and 2)			[14]
	$12,197 (≥ Grade 3)			
Caregiver costs (per month)			± 20%	
Progression-free	$1462			[17]
Progression	$2521			[17]
Best supportive care (per month)	$2754		± 20%	[16]
Utility				
Progression-free state	0.745	0.74	± 20%	[18, 19]
Progressive state	0.65	0.65	± 20%	[20]

*PFS* progression-free survival, *OS* overall survival, *BSC* best supportive care

^a^Drug acquisition cost for panitumumab of 400 mg/20 ml vial

^b^Drug acquisition cost for cetuximab of 100 ml 2 mg/ml vial

The cycle unit of the model was 1 month, with a half-month correction. The model considered a 61-year-old male as the base-case patient, to match the baseline patient characteristics of the ASPECCT trial. All dosage and other relevant parameters were based upon this base-case. The primary endpoint of the model is quality-adjusted life-years (QALYs). Other outputs included discounted and undiscounted life-months and costs. We calculated both incremental cost-effectiveness ratios (ICER) and incremental net monetary benefits (INMB).

### Transition probabilities

Median overall survival (OS) and median progression-free survival (PFS) of patients taking panitumumab and cetuximab were obtained directly from the ASPECCT trial. The decreasing exponential approximation of life expectancy (DEALE) method was used to calculate the monthly probabilities based on these medians. The DEALE method assumes that patients have a constant hazard of death throughout the time period being modeled [8, 9]. This assumption was appropriate in our case, given the short life expectancy of the patient population. In addition, the transition probabilities were adjusted for background all-cause mortality which can be obtained from the Actuarial Life Table of Social Security website, which in this case reflected the mortality rate of a 61-year-old US male.

### Direct costs

Direct costs considered by the model included the cost of drugs, administration, and management of adverse events (AEs) associated with treatment that was incurred during the progression-free state, and drug costs and other medical costs during the progressive state. All costs were converted into 2017 US dollars using the medical services component of the consumer price index.

#### Progression-free state

The prices of the two EGFR antibodies were obtained from the 2017 ASP Drug Pricing Files from Centers for Medicare and Medicaid Services, and we used ASP + 6% in calculating the drug acquisition cost [10]. Required doses of the two agents were calculated according to the prescribing information. Panitumumab is given at the dose of 6 mg/kg every 2 weeks, and cetuximab is given at 400 mg/m^2^ at the initial dosing and followed by 250 mg/m^2^ for every week. An alternative schedule (500 mg/m^2^ every 2 weeks) of cetuximab, which is more commonly used in clinical practice, was tested in the sensitivity analysis. The height and weight of the base-case needed to calculate the dose were obtained from CDC National Health Statistics Reports [11]. The cost of administration and other infusions was obtained from the Medicare Coding and Payment for Drug Administration Services under the Physician Fee Schedule [12]. Premedication drug costs and any related administration costs were also captured in the model.

The model considered costs for managing AEs with grade 3 or higher severity, except for infusion reactions where all grades were included. Since the reported incidence rate for grade 3 or higher AEs in the ASPECCT trial was very similar between the two treatment groups, the costs would be fully offset when calculating the incremental costs. Hence, the model only included AEs that differed between the two groups by over 1%, i.e. skin rash, hypomagnesaemia, hypokalemia and infusion reaction. Assumptions for managing AEs were based on recent published guidelines and literature [13–15].

#### Progressive state

The model considered the drug cost of regorafenib or TAS-102, and the cost of best supportive care after the disease progressed. Again, the drug costs were obtained from the Medicare ASP and fee schedule. As for the cost of best supportive care (BSC), Lang et al. estimated a lifetime, as well as phase-specific costs for CRC patients with different stages in the US [16]. For patients with metastatic CRC and those with an expected survival of less than 13-months, the annual BSC cost estimate was $31,980 after adjusting for inflation. This was applied in our model as total direct cost of the best supportive care in progressive state.

### Indirect costs

Caregiver’s time and lost alternative compensation due to caring for the mCRC patients were included in the model as indirect costs. Van Houtven et al. reported the estimated economic burden of informal caregivers of CRC patients during different phases [17]. Their study reported the cost of caring for patients during the initial phase (first year after diagnosis, not within 6 months of death), continuing phase (after 1 year, not within 6 months of death), and terminal phase (within 6 months of death) of disease. In our model, we assumed the caregiver costs in the progression-free state to be the cost of caring for patients in the continuing phase, which was estimated to be $24,423 over the accumulated 16.7 months after adjusting for inflation. This is justifiable because the patients in our model were receiving third- or further lines of chemotherapy, and should no longer be considered in the initial disease phase. The caregiver costs in the progressive disease state was assumed to be the cost of caring for terminal phase patients, which was reported as $17,645 over a 7-month period.

### Health state utilities

The utilities of the progression-free state for both panitumumab and cetuximab used in the model were reported from clinical trials. Bennett et al. analyzed patient report outcomes from a phase 3 clinical trial (NCT00339183) evaluating panitumumab plus FOLFIRI compared to FOLFIRI alone as second line therapy for mCRC [18]. The patients were all wild-type KRAS carriers and the HRQOL was measured by the EQ-5D Health State Index. The baseline EQ-5D was 0.769 in the panitumumab + FOLFIRI group, and according to the mixed model, the change from baseline score until disease progression was − 0.024. For cetuximab, we used utilities reported by patients using Health Utilities Index 3 (HUI-3) from a phase 3 trial evaluating cetuximab versus best supportive care among patients with chemo-refractory mCRC [19]. The mean of utilities assessed at different weeks until progression from the trial was used as the model input for cetuximab.

The utility of progressive state in the model was set to be same between the two arms, considering no more treatment toxicities after stopping EGFR inhibitor and both groups should develop similar disease patterns. The utility value was referenced to Ramsey et al. which has reported a HUI-3 utility of CRC survivors with less than 1-year expected survival [20].

### Sensitivity analysis

Both univariate sensitivity analysis and probabilistic sensitivity analysis were performed to evaluate the robustness of the model and address uncertainty in the estimation of model parameters. Survival data were varied over their 95% confidence interval reported from the trials; costs and utilities were varied within ± 20% of their baseline values. The alternative dose schedule of cetuximab was also tested.

## Results

Using all base-case parameters, panitumumab produced 7.8 months life-expectancy gain, 5.36 quality-adjusted life-months (QALMs) and 0.45 QALYs, at a cost of $66,006 per patient. While cetuximab produced similar clinical effectiveness of 7.9 months life expectancy, 5.42 QALMs and 0.45 QALYs, it costs $71,956 per patient, with an incremental cost of $5950. Thus, the base-case ICER is $1.25 millions per QALY for panitumumab relative to cetuximab. We also calculated incremental net monetary benefits (INMB) applying the willingness-to-pay threshold of $150,000, which was roughly three times the US GDP per capita according to the WHO criteria for cost-effectiveness thresholds [21]. Compared to cetuximab, the use of panitumumab yielded an expected gain of $5237 (Table 2).

**Table 2:** Base-case results

	Panitumumab	Cetuximab	Difference
Life-month gained	7.84	7.92	
Total QALM	5.36	5.42	
Total QALY	0.45	0.45	
Total costs	$66,006	$71,956	
Net monetary benefit	$1044	$ (4193)	
Cost-effectiveness ratio	$147,663	$159,281	
INMB	–	–	$5237
ICER	–	–	$1,251,775

*QALM* quality-adjusted life month, *QALY* quality-adjusted life year, *INMB* incremental net monetary benefit, *ICER* incremental cost-effectiveness ratio

**Fig. 1:**
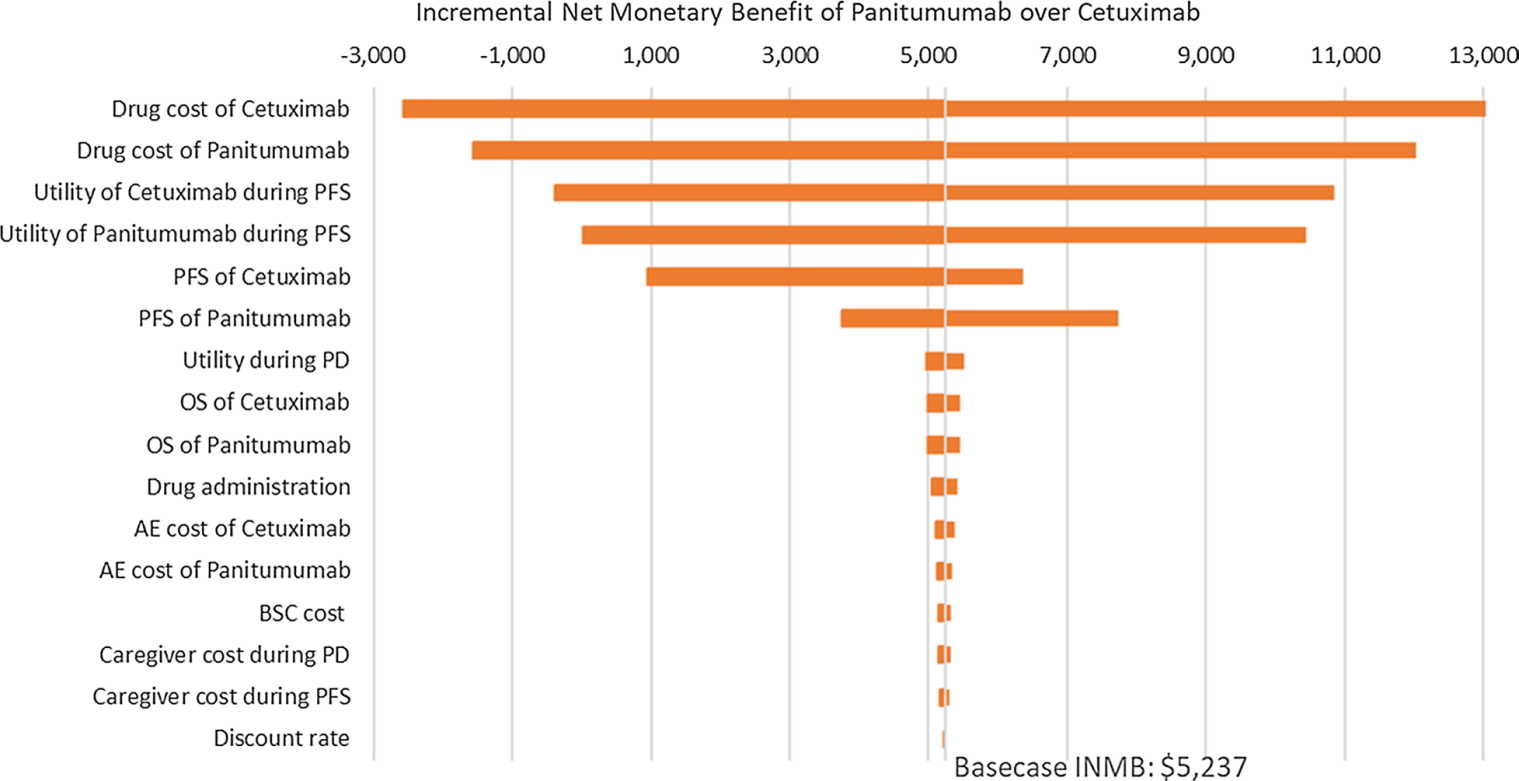
One-way sensitivity analysis tornado diagram. *PFS* progression-free survival, *PD* progression disease, *OS* overall survival, *BSC* best supportive care, *AE* adverse event

The results of univariate sensitivity analysis (Fig. 1) showed that in most scenarios tested, panitumumab had a positive INMB over cetuximab. Among all the parameters, the drug cost of cetuximab had the biggest impact on results. A price reduction of 13% for cetuximab would make the overall costs of the two treatment options equivalent. The model also showed sensitivity to the utilities of progression-free survival, and length of survival. Treatment costs of AEs, caregiver cost and best supportive cost all had minimal impact on the model. In addition, using the alternative dose schedule for cetuximab (i.e., 500 mg/m^2^ every 2 weeks) did not change the results significantly, resulting in an INMB of $2748 and ICER of $728,036 per QALY.

**Fig. 2:**
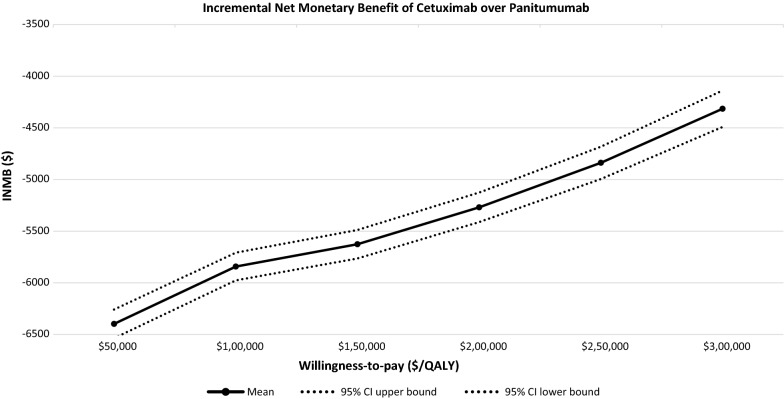
Probabilistic sensitivity analysis on INMB over willingness-to-pay

The results of probabilistic sensitivity analysis confirmed the finding from the univariate sensitivity analysis, showing that at various willingness-to-pay thresholds, cetuximab produces less net monetary benefit than panitumumab and the difference changes little when the threshold of willingness-to-pay increases (Fig. 2).

## Discussion

This analysis establishes that substitution of panitumumab for cetuximab will result in a $5237 cost saving per patient without any difference in the outcome. As stated earlier, 20% of patients with colorectal cancers are diagnosed with metastatic disease and 40% develop metastatic disease in their lifetime, and that 50% of patients are RAS wildtype and thus candidate for EGFR antibodies, thus the use of panitumumab for this population will lower the cost of care by more than 150 million dollars per year for payers.

Previous work has largely concluded that panitumumab and cetuximab, either as monotherapy or in combination, were not cost-effective when compared to the backbone chemotherapy of mCRC, which usually contains FOLFOX, FOLFIRI, and bevacizumab [22–25]. There are no studies specifically comparing the economic value of EGFR antibodies in mCRC patients. The existence of two EGFR antibodies with comparable efficacy in a large head-to-head clinical trial provides compelling reasoning for choosing one over the other based on their relative economic value. This may justify choosing the agent with the best economic value and providing the opportunity for market competition, and hopefully, price reduction. The economic comparison of therapies that are similar in effectiveness should be of interest to oncologists, payers, and cancer patients, and may help inform decisions about clinically comparable agents with different costs.

Recently, the manufacturer of panitumumab has done a cost-minimization analysis of panitumumab versus cetuximab in the first-line treatment setting [26]. Their model only captured the direct cost component but did not account for the difference in other aspects, such as infusion time. Our model has taken a traditional approach of conducting cost-effectiveness analysis by considering clinical effectiveness, incidence of adverse events, both direct and indirect costs, and health utilities. The clinical effectiveness data from the head-to-head trial has provided a strong foundation for conducting this comparative effectiveness study. While the trial showed a similar effectiveness and safety profile for the two agents, our results indicate that panitumumab can lower the cost of mCRC care from a US societal perspective, without compromising patient quality of life. Drug costs as well as other direct costs in our main analysis are based on the Medicare prices. The same results were obtained using cost data from the Veterans Affairs Federal Supply Schedule, a publicly available source for cost of drugs recommended by the most recent US Panel on Cost Effectiveness in Health (data not shown).

Several limitations to our model should be noted. First, assumptions have been made in calculating the cost of treating AEs. For patients experiencing severe skin toxicities, we converted the inpatient care cost per event into monthly costs by dividing the per event cost by treatment duration. We assumed very severe skin toxicity would interrupt the treatment temporarily and EGFR inhibitors may be continued among these patients based on NCCN guidelines [4]. Also, it is difficult to project the monthly frequencies of outpatient visits due to skin toxicity, which creates some uncertainties in the cost estimation for treating AEs. However, as the model sensitivity analysis shows, it is unlikely that these uncertainties would have any substantial impact on the results. Second, health utilities were collected from different trials using different instruments. For the utilities of panitumumab during the progression-free state, we used the reported EQ-5D from mCRC patients receiving the drug in combination with FOLFIRI as a second-line treatment which was compared with patients receiving FOLFIRI alone. However, the utilities of cetuximab during the progression-free state were assessed using HUI-3 among chemo-refractory patients receiving cetuximab alone. Despite the fact that patients who receive EGFR inhibitors may experience similar drug-related adverse events, the different instruments used may have produced slightly different results. In addition, this model takes the US societal perspective where utilities generated from the general public would be most ideal, however, the utilities actually used in this model were collected from patients. Nevertheless, any uncertainties around the health utilities should have been captured in the sensitivity analysis and would not meaningfully alter our final conclusions. Third, while the selected adverse events are the most commonly reported among patients receiving EGFR inhibitors, the incidence rate of grade 3 and 4 AEs from the ASPECCT trial may not represent what happens in real clinical settings. For example, the incidence rates of grade 3–4 hypomagnesaemia from the ASPECCT trial were 7.06% in the panitumumab arm and 2.58% in the cetuximab arm. However, in other sources, the incidence rate may range between 6% and 47%, depending on the length of therapy received [27]. Last but not least, the model calculated the drug cost based on dose received by the base-case of a 61-year-old male, and didn’t account for any vial wastage. Again, the difference between no-vial-wastage and with-vial-wastage is about 10% [23] and we have tested 20% cost differences in the model sensitivity analysis.

## Conclusion

We used data reported in ASPECCT to evaluate the cost-effectiveness of panitumumab and cetuximab as monotherapy in chemo-refractory mCRC patients with wild-type KRAS from a US societal perspective. We demonstrate that substitution of cetuximab with panitumumab in this patient population will result in substantial cost saving. Physicians and payers should consider these results in oncology drug formulary choice and contracting.
